# Effects of Danshensu and Salvianolic Acid B from *Salvia miltiorrhiza* Bunge (*Lamiaceae*) on Cell Proliferation and Collagen and Melanin Production

**DOI:** 10.3390/molecules19022029

**Published:** 2014-02-13

**Authors:** Yi-Shyan Chen, Shu-Mei Lee, Ying-Ju Lin, Shu-Hua Chiang, Chih-Chien Lin

**Affiliations:** 1 Department of Cosmetic Science, Providence University, 200 Chung-Chi Road, Shalu, Taichung 43301, Taiwan; E-Mail: yishyan@gm.pu.edu.tw; 2 Department of Cosmetic Science and Management, Mackay Medicine, Nursing and Management College, 92 Shengjing Road, Beitou, Taipei 11260, Taiwan; E-Mail: s107@eip.mkc.edu.tw; 3 Department of Medical Research, China Medical University Hospital, 2 Yuh-Der Road, Taichung 40447, Taiwan; E-Mail: yjlin.kath@gmail.com; 4 School of Chinese Medicine, China Medical University, 91 Hsueh-Shih Road, Taichung 40402, Taiwan; 5 Department of Food and Beverage Management, Taiwan Hospitality and Tourism College, 268 Chong-Hsing St., Feng-Shan Village, Shou-Feng County, Hualien 974, Taiwan

**Keywords:** collagenesis, danshensu, melanogenesis, salvianolic acid B, *Salvia miltiorrhiza*

## Abstract

Danshensu (DSU) and salvianolic acid B (SAB) are the primary water-soluble compounds of *Salvia miltiorrhiza* Bunge (*Lamiaceae*). In this study, we analyzed the effects of DSU, SAB and a *S. miltiorrhiza* extract (SME) on cell proliferation. Additionally, the effects of DSU and SAB on collagen synthesis in Detroit 551 human normal fibroblast cells and on melanin production in B16 melanoma cells were verified. The results demonstrated that SME can enhance the proliferation of Detroit 551 cells and that this boost may be caused by DSU and SAB. This research showed that SME, DSU and SAB all have the ability to increase the production of collagen in Detroit 551 cells. The results also confirmed that DSU and SAB can attenuate the α-MSH-stimulated melanin production of B16 cells by inhibiting tyrosinase activity. Therefore, SME, DSU and SAB each have the potential to be utilized as active ingredients in wound healing or cosmetic treatments. In the future, DSU and SAB could also be used as functional components for treating hyperpigmentation.

## 1. Introduction

**Figure 1:**
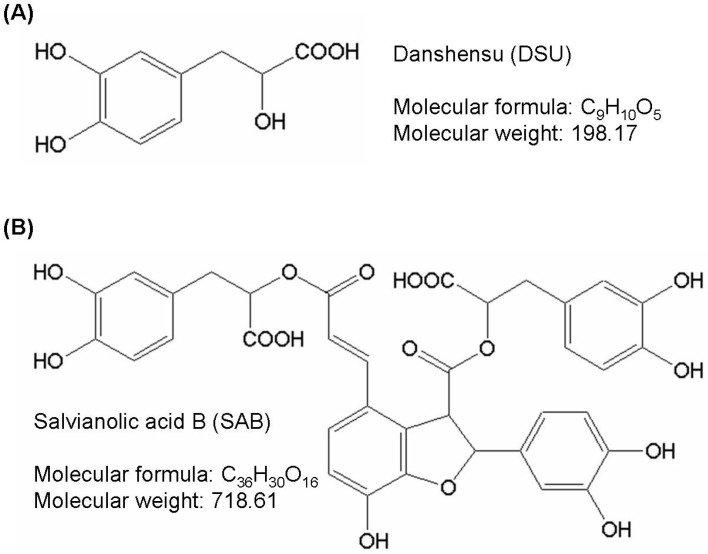
The chemical structures of (**A**) danshensu and (**B**) salvianolic acid B.

*Salvia miltiorrhiza* Bunge (*Lamiaceae*), generally known as danshen, is an important and widely used medicinal plant in Traditional Chinese Medicine (TCM). It is used in many countries, including China, Korea and Japan [1]. According to the principles of TCM, the dried root of *S. miltiorrhiza* can be used to promote blood flow and to resolve blood stasis [2]. The beneficial effects of *S. miltiorrhiza* on the cardiovascular system have been broadly demonstrated [3,4]. The chemical components of an extract of *S. miltiorrhiza* are classified into two main groups: water-soluble (hydrophilic) phenolic compounds and lipid-soluble (nonpolar, lipophilic) diterpenoidal compounds. These active components of *S. miltiorrhiza*, including tanshinones, tanshinlactone, salvianolic acids and danshensu, have potent antioxidant, antimicrobial, anticancer and cardioprotective functions [5,6,7]. Danshensu (DSU), salvianolic acid A (SAA) and salvianolic acid B (SAB) are phenolic caffeic acid derivatives, and they are the primary water-soluble compounds of *S. miltiorrhiza*. Among them, danshensu and salvianolic acid B are present in the highest concentrations [8]. Their structures are shown in Figure 1. Danshensu is a relatively simple phenolic acid found in the *S. miltiorrhiza* extract that exhibits cardiovascular protective effects. Additionally, danshensu acts on human umbilical vein endothelial cells to protect against injuries and inhibit the production of ROS, effects that have been established by several studies [7,9,10]. The therapeutic potential of salvianolic acid B in hepatic protection, neural protection and cancer treatment has been proposed in recent years, although the compound’s greatest clinical impact is in cardiovascular protection [11,12,13,14]. However, the effects of danshensu and salvianolic acid B on the regulation of collagenesis and melanogenesis have not been fully studied.

The synthesis of melanin in melanocytes is catalyzed by melanogenic enzymes, including tyrosinase, tyrosinase-related protein 1 (TRP-1) and tyrosinase-related protein 2 (TRP-2). These enzymes play critical roles in melanin production via the hydroxylation of tyrosine into dihydroxyphenylalanine (DOPA) and the further oxidation of DOPA into DOPAquinone. The increased activity of these enzymes, caused by stimulative factors such as ultraviolet (UV) light and chronic inflammation, may lead to hyperpigmentation. Therefore, melanogenesis inhibitors can be used to treat hyperpigmentation in skin [15,16].

Collagens are the most abundant proteins in mammals; they play structural roles, contributing to the mechanical properties, organization and shape of tissues. Collagens interact with cells to regulate their proliferation, migration, and differentiation [17]. In the collagen superfamily, type I collagen is the most rich extracellular matrix protein and is essential for the mechanical strength of tissues [18]. Thus, the regulation of collagen production has the potential to treat the tissue disorders that are related to collagens.

In this study, we analyzed the effects of danshensu and salvianolic acid B on collagen synthesis in Detroit 551 normal fibroblast cells and on melanin production in B16 melanoma cells. Additionally, the effects of danshensu, salvianolic acid B and a *S. miltiorrhiza* extract on cell proliferation were tested.

## 2. Results and Discussion

### 2.1. Effects of SME, DSU and SAB on the Proliferation of Fibroblast Cells

**Figure 2:**
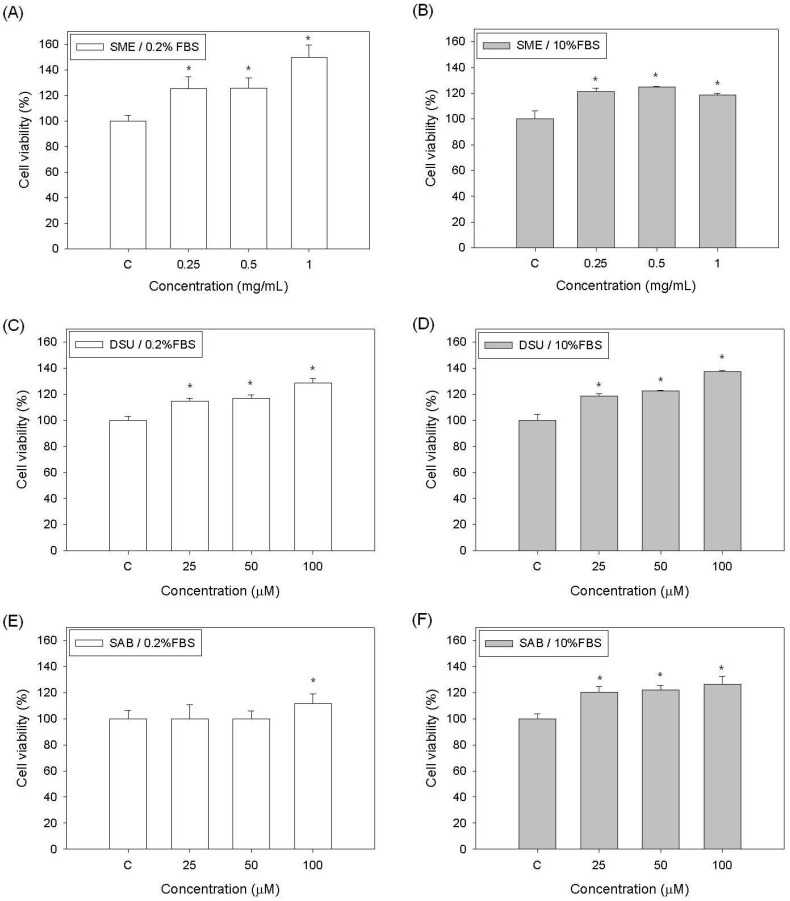
The effects of a *S. miltiorrhiza* extract (SME), danshensu (DSU) and salvianolic acid B (SAB) on the cell proliferation of Detroit 551 cells: (**A**) SME in 0.2% FBS; (**B**) SME in 10% FBS; (**C**) DSU in 0.2% FBS; and (**D**) DSU in 10% FBS; (**E**) SAB in 0.2% FBS, and (**F**) SAB in 10% FBS. Each value represents the mean ± SE (*n* = 3). * *p* < 0.05, compared with the control.

For the preparation of *S. miltiorrhiza* extract (SME), the dehydrated *S. miltiorrhiza* leaves were homogenized and then extracted with water at room temperature for 30 min. The prepared SME should contain plenty of water-soluble (hydrophilic) phenolic compounds, including danshensu (DSU) and salvianolic acid B (SAB). In this study, we first evaluated the effects of DSU, SAB and SME on the growth of Detroit 551 human normal fibroblast cells. The results are shown in Figure 2. As seen in Figure 2A,B, the viability of the Detroit 551 cells in both the low (0.2%) and normal (10%) FBS conditions increased when the cells were treated with different concentrations of SME. The enhanced cell viability of the group treated with 1 μg/mL SME in 0.2% FBS was approximately 150% of the control, the highest viability level observed (Figure 2A). Although the cell viabilities of the SME treated group in 10% FBS were only approximately 120% of the control, the cell proliferation-enhancing activity of SME in that condition was still observed (Figure 2B). When treated with DSU, the increased viabilities of the Detroit 551 cells in both FBS conditions were similar to those of the SME groups. Additionally, the increased levels observed in the DSU groups with either 0.2% or 10% FBS were nearly the same (Figure 2C,D). When treated with 100 μM DSU, the cell viability increased to 137% of the control (Figure 2D). Therefore, our results demonstrated that SME and DSU can improve the cell proliferation of Detroit 551 cells in both 0.2% and 10% FBS.

The growth of cells treated with SAB was only enhanced in 10% FBS. Under those conditions, the cell viability levels were approximately 120% of the control (Figure 2F). However, under 0.2% FBS conditions, treatment with 100 μM SAB enhanced the growth of the Detroit 551 cells but not significantly (Figure 2E). Our results established that SME has a growth factor (GF)-like ability to enhance the growth of human normal fibroblast cells and that this effect might be caused by the components DSU and SAB. Earlier study has demonstrated that several human cancer cell lines exhibited decreased cell viability when exposed to 125 µM SAB for 24 h [11]. Moreover, our results also revealed that the effects of DSU and SAB on cell proliferation will be attenuated when the concentrations of are higher than 200 µM (data not shown). Thus, in this study, we selected 100 µM as the highest concentration for our experiments.

Many functional materials have demonstrated an ability to enhance cell proliferation. For wound healing purposes, a cell growth-enhancing activity may help rehabilitate cut tissues in a shorter time. Additionally, cell activation is a key property for cosmetic skin morphogenesis [19,20,21]. Therefore, SME, DSU and SAB have the potential to be used as functional ingredients in wound healing or cosmetic treatments.

### 2.2. Effects of DSU and SAB on the Proliferation of EGF-Treated Fibroblast Cells

**Figure 3:**
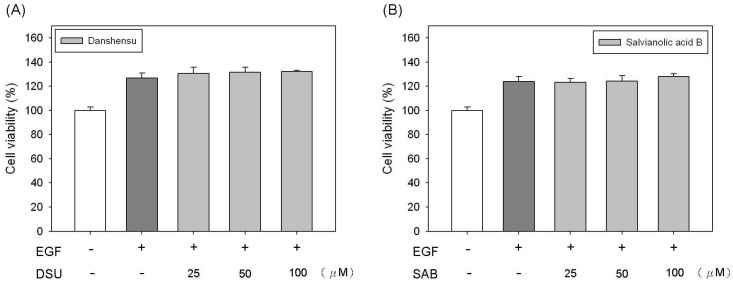
The effects of (**A**) danshensu (DSU) and (**B**) salvianolic acid B (SAB) on the cell proliferation of epidermal growth factor (EGF)-treated Detroit 551 cells in 0.2% FBS. Each value represents the mean ± SE (*n* = 3). * *p* < 0.05, compared with the control.

To further confirm the functions of DSU and SAB on the proliferation of fibroblast cells, we tested the cell viability of Detroit 551 cells co-treated with DSU or SAB and human epidermal growth factor (EGF). The results are shown in Figure 3. Our results demonstrated that DSU and SAB cannot enhance the cell proliferation of EGF-treated Detroit 551 cells at any of the concentrations used. The cell viabilities of all EGF-treated groups are approximately 125% to 130% of the control (Figure 3). Therefore, we can suggest that the effects of DSU and SAB on the proliferation of fibroblast cells are related to the signaling pathway of EGF.

### 2.3. Effects of SME, DSU and SAB on the Collagen Production of Fibroblast Cells

**Figure 4:**
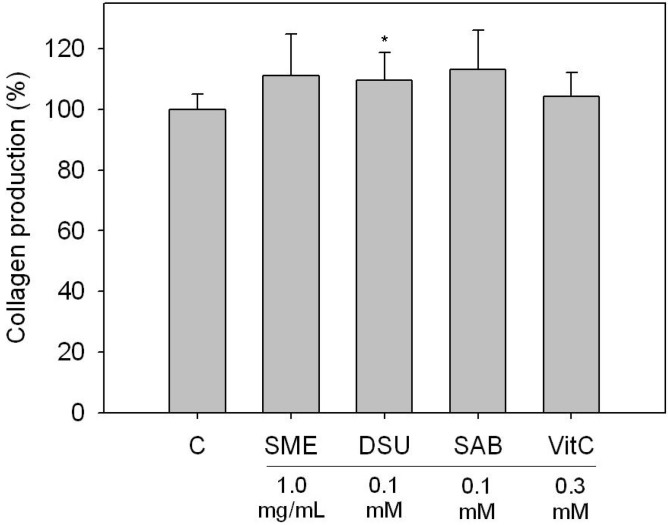
The effects of a *S. miltiorrhiza* extract (SME), danshensu (DSU) and salvianolic acid B (SAB) on the collagen production of Detroit 551 cells. C is the control group and VitC is vitamin C. Each value represents the mean ± SE (*n* = 3). * *p* < 0.05, compared with the control.

Due to the cell proliferation-enhancing effect of SME, DSU and SAB on fibroblast cells, we hypothesized that the compounds could also enhance the production of collagen in fibroblast cells. Therefore, we analyzed the collagen levels of Detroit 551 cells treated with SME, DSU and SAB, and the results are shown in Figure 4. Our experiments revealed that all of the tested SME, DSU and SAB samples could increase collagen production in Detroit 551 cells. The increased collagen levels in the SME, DSU and SAB treated Detroit 551 cells were all approximately 110% of the control (Figure 4). Our assay specifically examined the quantities of type I through type V collagen. Although the increased collagen levels were not quite as high as 110% after 24 h treatment, we still believe that SME, DSU and SAB have the ability to improve the production of collagen in fibroblast cells. This is because the effects of SME, DSU and SAB on collagen production were equivalent to those of the positive control (vitamin C, 0.3 mM). Collagen expression is mainly regulated by the transforming growth factor beta (TGF-β)/Smads signaling pathway in fibroblast cells. Moreover, the mitogen activated protein kinase (MAPK) signaling pathway is necessary for receptor tyrosine-kinase induced transcription of TGF-β gene [22]. Therefore, we can propose that the activation of collagen production in SME-, DSU- and SAB-treated Detroit 551 cells were associated with their cell growth-enhancing functions.

### 2.4. Inhibition effects of DSU and SAB on Tyrosinase Activity

**Figure 5:**
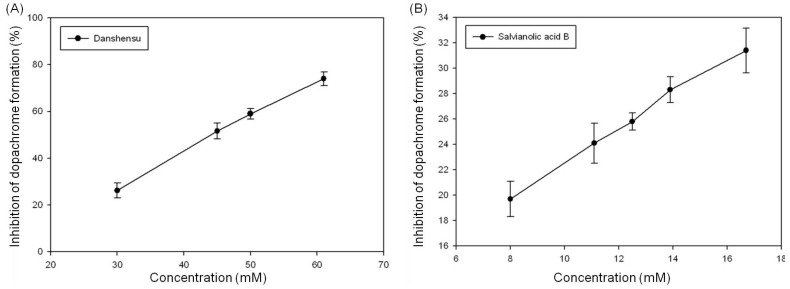
The inhibition effects of (**A**) danshensu and (**B**) salvianolic acid B on tyrosinase activity. Each value represents the mean ± SE (*n* = 3).

Because the chemical structure of DSU is similar to that of DOPA, we hypothesized that DSU and SAB might have the ability to inhibit tyrosinase activity. The results of this experiment are shown in Figure 5.

We found that the inhibition of dopachrome formation is increased with increasing DSU concentrations. An inhibition rate of 74% can be achieved with a DSU concentration of 60 mM (Figure 5A). The calculated IC_50_ value of DSU on dopachrome formation inhibition is 44.1 ± 1.1 mM. In contrast, Figure 5B shows that although SAB can suppress the inhibition of dopachrome formation in a dose-dependent manner, the suppression ratio only reaches approximately 31% at a concentration of 17 mM (the highest solubility of SAB in water). Ultimately, our results demonstrated that DSU and SAB both have the ability to inhibit the activity of tyrosinase, though DSU is more effective than SAB.

### 2.5. Inhibition Effects of DSU and SAB on Melanin Production in α-MSH-Stimulated Melanoma Cells

**Figure 6:**
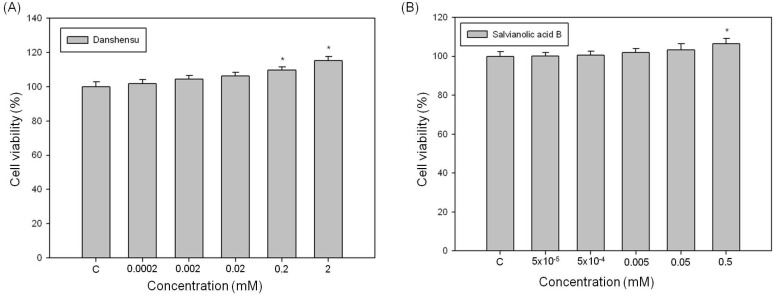
The effects of (**A**) danshensu and (**B**) salvianolic acid B on the cell viability of B16 melanoma cells. Each value represents the mean ± SE (*n* = 3). * *p* < 0.05, compared with the control.

Because the tyrosinase used in our experiment was isolated from mushrooms, the inhibition effects of DSU and SAB on melanin production were further confirmed in a cell based assay model. Therefore, we tested the inhibition effects of DSU and SAB on melanin production in α-MSH-stimulated B16 melanoma cells. First, we verified the effect of DSU and SAB on the growth of the B16 cells. Those results are shown in Figure 6, and neither DSU nor SAB showed cytotoxicity in B16 cells at the tested concentrations. Moreover, at higher concentrations, DSU and SAB can slightly increase the viability of B16 cells. Although the B16 cell growth-enhancing effects of DSU and SAB are not notable when compared with those of Detroit cells, they were established in our experiments (Figure 6A,B).

**Figure 7:**
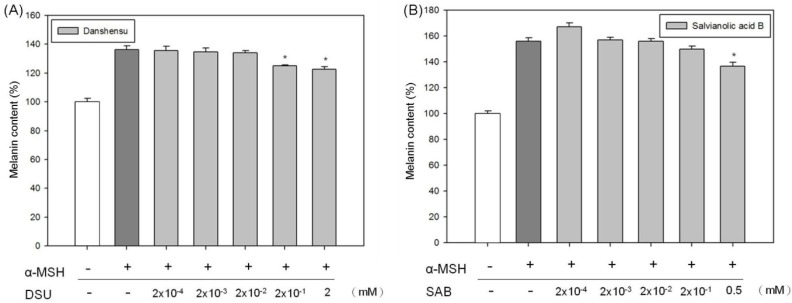
The inhibition effects of (**A**) danshensu (DSU) and (**B**) salvianolic acid B (SAB) on melanin production in α-MSH-stimulated B16 cells. Each value represents the mean ± SE (*n* = 3). * *p* < 0.05, compared with the control.

Subsequently, we verified the inhibition effects of DSU and SAB on melanin production in α-MSH-stimulated B16 melanoma cells, and those results are shown in Figure 7. Under α-MSH stimulation, the melanin content was increased to approximately 150% of the control (untreated B16 cells). Treatment with high concentrations of DSU and SAB decreased the α-MSH-stimulated melanin levels. A concentration of 2 mM DSU reduced the α-MSH-stimulated melanin content by 15% (Figure 7A). Treatment with 0.5 mM SAB attenuated the α-MSH-stimulated melanin content by approximately 20% (Figure 7B). Therefore, both DSU and SAB can suppress melanin production in α-MSH-stimulated B16 cells. Moreover, the effects of the compounds on melanin production are different from their impact on tyrosinase activity. This difference may have occurred because the tyrosinase used came from mushrooms, and tyrosinases from different species have diverse protein structures that can lead to dissimilar results.

The synthesis of melanin in melanocytes can be induced by numerous factors, including α-MSH, cyclic adenosine monophosphate (cAMP)-elevating agents (such as isobutylmethylxanthine and forskolin) and UV light [23,24]. Finding agents to regulate melanin synthesis is important for the treatment of hyper- or hypo-pigmentation [25,26,27]. Our previous study found that SME can strongly enhance tyrosinase activity and melanin production [28]. Thus, although DSU and SAB are the major components of the water extract of *S. miltiorrhiza*, SME and DSU/SAB can have totally opposite effects on biological functions. For that reason, we suggest that some other functional components in SME strongly increase melanin production in melanoma cells; this effect must be stronger than the inhibition of melanin production provided by DSU and SAB. Regardless, our results continue to demonstrate that DSU and SAB have the potential to treat hyperpigmentation.

## 3. Experimental

### 3.1. Materials

*Salvia miltiorrhiza* was provided by WinPower Technology Co., Ltd. (Kaohsiung, Taiwan). Danshensu and salvianolic acid B were purchased from Fusol Material Co., Ltd. (Tainan, Taiwan). Human recombinant epidermal growth factor (EGF) was purchased from ProSpec Inc. (Ness Ziona, Israel). Vitamin C, Triton X-100, mushroom tyrosinase, L-tyrosine, α-melanocyte stimulating hormone (α-MSH), dimethyl sulfoxide (DMSO) and other chemicals were purchased from Sigma-Aldrich (St. Louis, MO, USA). Dulbecco’s modified Eagle’s medium (DMEM), α-modified essential medium (α-MEM), fetal bovine serum (FBS), L-glutamine, penicillin-streptomycin and trypsin ethylenediaminetetraacetic acid (trypsin EDTA) were purchased from Invitrogen Life Technologies (Carlsbad, CA, USA). The compound 3-(4,5-dimethylthiazol-2-yl)-2,5-diphenyltetrazolium bromide (MTT) was purchased from Affymetrix/USB (Cleveland, OH, USA). The deionized distilled water (ddH_2_O) used in solutions and buffers was purified with a Milli-Q system (Millipore, Bedford, MA, USA).

### 3.2. Preparation of Extracts

The leaves of *S. miltiorrhiza* were cleaned with pure water. After cleaning, the prepared leaves were dried by airing. The dehydrated *S. miltiorrhiza* leaves were homogenized and then extracted with cold water at 25 °C for 30 min. The collected supernatant was filtered by 0.45 μm filters to remove any debris. The filtered *S. miltiorrhiza* extract (SME) was freeze-dried and stored at 4 °C prior to use [28].

### 3.3. Cell Culture and MTT Assay

The Detroit 551 normal fibroblast cells (BCRC 60118) and the B16 melanoma cells (BCRC 60031) were purchased from the Food Industry Research and Development Institute (FIRDI, Hsinchu, Taiwan). The Detroit 551 and B16 cells were cultured in α-MEM and DMEM, respectively, and then supplemented with 10% FBS, 2 mM glutamine, 100 mg/mL streptomycin and 100 U/mL penicillin. The cells were maintained in a humidified incubator with 5% CO_2_ at 37 °C, and they were sub-cultured every 3 to 4 days to maintain logarithmic growth. For the cell viability assays, cells were seeded in a 96-well plate at a density of 5 × 10^3^ cells/well. After 24 h of incubation, different concentrations of the test compounds or EGF (0.2 μg/mL) were added to each well of the plate, and then the plate was incubated for an additional 24 h. Cell viability was determined via an improved MTT assay [29].

### 3.4. Collagen Assay

The total amount of soluble collagen was assessed using a Sircol Collagen Assay Kit according to the manufacturer's instructions (Biocolor, Carrickfergus, Northern Ireland, UK). In the collagen assay, Detroit 551 cells (6 × 10^5^ cells/well) were incubated in a 6-well plate with various concentrations of the test compounds for 24 h, and then the cells were collected and lysed using 300 μL PBS with 1% Triton X-100. The samples were centrifuged (12,000 rpm for 10 min), and each 100 μL of either the cell sample or a collagen standard was mixed with 1 mL of Sircol dye for 30 min and then centrifuged at 10,000 rpm for 5 min to separate the collagen-dye complex from the supernatant. The collagen-dye complex was dissolved in 1 mL of Sircol alkali reagent and vortexed. Vitamin C was used as a control. The absorbance of the solution was examined at 540 nm. The quantity of collagen was calculated from the collagen standards and expressed as a percentage of the control [30].

### 3.5. Tyrosinase Activity Assay

For the tyrosinase activity assay, 60 μL samples of different concentrations of the test compounds were mixed with 100 μL of 1 mM L-tyrosine in phosphate buffer solution (pH 6.8). Then, 40 μL of mushroom tyrosinase solution (100 units/mL) was added to the mixture and incubated for 25 min at 37 °C. A spectrophotometric analysis was performed at 475 nm, and the inhibition of dopachrome formation was calculated as an inhibition percentage [31].

### 3.6. Melanin Content Assay

B16 melanoma cells (2 × 10^5^ cells/well) were incubated in 6-well plates with various concentrations of the test compounds. The cells were co-treated with 100 nM α-MSH for 24 h. After this treatment, the cells were dissolved in 120 μL of 1 N NaOH for 1 h at 65 °C to solubilize the melanin. The total amount of melanin in each cell suspension was determined by recording the absorbance at 405 nm. The melanin content was calculated and corrected for the cell number [32].

### 3.7. Statistical Analysis

All analytical measurements were performed in triplicate. The results were analyzed using Student’s *t*-test and were expressed as the mean ± standard error for each measurement. *p*-values less than 0.05 were considered to be significant.

## 4. Conclusions

**Figure 8:**
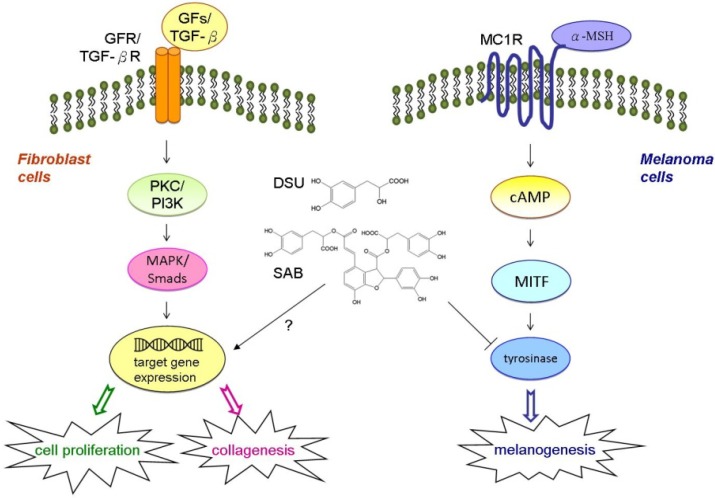
Proposed mechanisms of danshensu (DSU) and salvianolic acid B (SAB) on cell proliferation and collagen and melanin production.

In summary, our experiments demonstrated that SME has a growth factor-like ability to enhance the proliferation of Detroit 551 fibroblast cells and that this effect may be caused by DSU and SAB. Additionally, SME, DSU and SAB all have the ability to increase the production of collagen in Detroit 551 cells. Our results also confirmed that DSU and SAB can suppress melanin production in α-MSH-stimulated B16 melanoma cells through the inhibition of tyrosinase activity. The proposed mechanisms of DSU and SAB on cell proliferation and collagen and melanin production are shown in Figure 8. Therefore, SME, DSU and SAB have the potential to be used as active ingredients for wound healing and cosmetic treatments. Moreover, DSU and SAB also have the potential to treat hyperpigmentation.
